# The Effect of Blood Pressure on Cognitive Performance. An 8-Year Follow-Up of the Tromsø Study, Comprising People Aged 45–74 Years

**DOI:** 10.3389/fpsyg.2020.00607

**Published:** 2020-04-21

**Authors:** Knut Hestad, Knut Engedal, Henrik Schirmer, Bjørn Heine Strand

**Affiliations:** ^1^Department of Health Studies, Inland Norway University of Applied Sciences, Elverum, Norway; ^2^Department of Research, Innlandet Hospital Trust, Ottestad, Norway; ^3^Norwegian National Advisory Unit on Ageing and Health, Vestfold County Hospital Trust, Tønsberg, Norway; ^4^Department of Cardiology, Akerhus University Hospital, Lørenskog, Norway; ^5^Institute of Clinical Medicine, University of Oslo, Oslo, Norway; ^6^Institute of Clinical Medicine, UiT-The Arctic University of Norway, Tromsø, Norway; ^7^Department of Chronic Diseases and Ageing, Norwegian Institute of Public Health, Oslo, Norway

**Keywords:** blood pressure, cognitive performance, aging, sex differences, dementia

## Abstract

**Background:**

The relationship between blood pressure (BP) and cognition is complex were age appears to be an intervening variable. High and low BP have been associated with cognitive deficits as part of the aging process, but more studies are needed, especially in more recent birth cohorts.

**Methods:**

The study sample comprised 4,465 participants, with BP measured at baseline in the Tromsø Study, Wave 6 in 2007–2008 (T0), and cognition assessed at follow-up 8 years later, in 2015–2016 in Tromsø Study 7 (T1). Age at T0 was 45–74 years, and at T1 it was 53–82 years. Cognition was assessed with three tests: The Mini Mental State Examination (MMSE), the Digit Symbol Test, and the Twelve-word Test. The associations between BP and cognition were examined specifically for age and sex using linear regression analysis adjusted for baseline BP medication use, education and body mass index (kg/m^2^).

**Results:**

BP was associated with cognition at the 8-year follow-up, but the association differed according to age and sex. In men, higher systolic blood pressure (SBP) and diastolic blood pressure (DBP) at a young age (45–55 years of age) was associated with poorer cognition; the association was reversed at older ages, especially for those above 65 years of age. In women, the associations were generally weaker than for men, and sometimes in the opposite direction: For women, a higher SBP was associated with better cognition at a younger age and higher SBP poorer cognition at older ages – perhaps due to an age delay in women compared to men. Digit Symbol Test results correlated best with BP in a three-way interaction: BP by age by sex was significant for both SBP (*p* = 0.005) and DBP (*p* = 0.005).

**Conclusion:**

Increased SBP and DBP at the younger age was clearly associated with poorer cognitive function in men 8 years later; in women the associations were weaker and sometimes in the opposite direction. Our findings clearly indicate that interactions between age and sex related to BP can predict cognitive performance over time. Men and women have different age trajectories regarding the influence of BP on cognition.

## Introduction

The association between blood pressure (BP) and cognitive function is a complex one. It has been suggested that both high and low BP may be predictors of cognitive deficits in later years (Qiu et al., 2005), and it seems that the timing of the BP measurement is a key variable. As the population ages, an increase in mean BP has been observed – particularly a rise in systolic BP (SBP) (Franklin, 2006; McEniery et al., 2016). A recent study has shown that for those born in 1960 and later, however, BP does not increase with age (Hopstock et al., 2015).

High BP in midlife is a risk factor for stroke and for dementia arising from Alzheimer’s Disease (AD) and cerebrovascular disorders (Launer et al., 1995; Kilander et al., 1998; Swan et al., 1998a, b; Kivipelto et al., 2001; Yamada et al., 2003; Whitmer et al., 2005; Kimm et al., 2011; Joas et al., 2012). Skoog et al. (1996) suggest that high BP in midlife harms the brain, which thereafter results in BP decline and cognitive impairment. This conclusion fits with several findings of low BP later in late life being related to cognitive impairment and dementia (Hestad et al., 2005; Gabin et al., 2017). Furthermore, it has been shown that both high and declining DBP are associated with global brain atrophy, as indicated by magnetic resonance imaging (Heijer et al., 2003). However, the brain has an autoregulation system that may compensate for change in blood pressure, but if big changes occur abruptly, this autoregulation system may not be able to compensate. Joas et al. (2012) analyzed women only, in a longitudinal study, and concluded that high systolic pressure may be an indicator of large artery stiffness with an increased risk of dementia, whereas diastolic pressure could be a marker of peripheral resistance, which may be related to white matter lesions. Hypertension may start a degenerative process and in the long run result in brain damage and a decline in blood pressure, which is seen in people before development of dementia symptoms (Joas et al., 2012). Thus, both high pressure related to arterial stiffness and the later low pressure may result in reduced oxygen supply to the brain, which is needed for efficient cognitive performance.

However, two meta-analyses revealed no association between hypertension and AD (Guan et al., 2011; Power et al., 2011), although a large Hawaiian cohort study showed a correlation between high BP in midlife and cognitive dysfunction due to dementia of mild degree (Launer et al., 1995; Launer et al., 2000). Another US study concluded that midlife, but not late-life elevated SBP, was associated with cognitive decline (Gottesman et al., 2014). In line with this finding, in the Whitehall II cohort study, midlife SBP as low as 130 mmHg and higher was associated with increased risk of dementia, whereas no such association was seen for late-life SBP (Abell et al., 2018). Hebert et al. (2004), who studied participants 65 and older, found no association between elevated BP and cognitive decline, a finding that may reflect the age of their participants, who may have been too old to demonstrate an association.

Few studies have examined the relationship between high BP and cognition; most studies have examined dementia as the outcome of high BP. Thus, we sought to examine whether baseline BP over an 8-year period was associated with impaired cognition and if this association differed between age groups and sexes in a population based study.

Men and women seem to have different developmental stories regarding blood pressure regulations during life (Joyner et al., 2016). Women also have different patterns regarding incident of myocardial infarction with lower rates for women at least until 95 years of age compared to men (Barrett-Connor, 2013).

We also wanted to examine whether the use of antihypertensive drugs attenuated any observed association.

## Methods

### Study Population

The Tromsø Study is a longitudinal population-based multipurpose study initiated in 1974 with surveys and examinations to examine lifestyle-related diseases. Earlier participants were invited to join the study and new participants were recruited in 1979, 1986, 1994, 2001, 2007, and 2015 (Jacobsen et al., 2012; Eggen et al., 2013). In our study, data from the last two study waves are used: Tromsø Study Wave 6 (our baseline) and Wave 7 (our follow-up). The Tromsø study has a complex sampling design, with an attendance rate of 66% in Tromsø 6 and 65% in Tromsø 7^1^. Hereafter, to reflect our study design, Tromsø 6 is denoted T0 and Tromsø 7 is denoted T1, with BP measured at baseline in T0 and its association with cognition at follow-up in T1.

Our study sample comprised 4,465 Tromsø study participants (Table 1), born between 1933 and 1962, with baseline SBP and DBP measurements at T0 when participants were 45–74 years old; their cognition was assessed at follow-up at T1, when they were 53–82 years old. Thus, to be included in our study, participants must have had BP measurements at T0 and a valid cognitive test score at T1.

**TABLE 1:** Flow-chart, study sample were we also included one of the cognitive tests.

	N yes	N no		Baseline characteristics at Tromsø Wave 6 (T0)					
			Age, mean (SD)	Women, (%)	Education, (% high)	Poor self-rated health (%)	SBP, mmHg (SD)	BMI, kg/m^2^ (SD)	Digit Symbol, mean (SD)**
0. Invited to baseline examination, 45–74 years, T0	12,054								
1a. Did not participate in T0		3,241							
1b. Participated in T0	8,813		59.9 (7.8)	52	36	35	137.7 (21.9)	27.1 (4.3)	40.3 (12.6)
2a. No valid BP measurement in T0		34							
2b. Valid BP measurements in T0	8,779		59.9 (7.8)	52	36	35	137.7 (21.9)	27.1 (4.2)	40.3 (12.6)
3a. Not invited, died or emigrated before T1		416	65.1 (6.4)	40	30	56	143.3 (24.1)	26.9 (4.3)	33.0 (11.9)
3b. Invited to T1, Phase I	8,363		59.7 (7.8)	52	36	34	137.4 (21.7)	27.1 (4.2)	40.7 (12.5)
4a. Did not participate in T1, Phase I		1,895	61.0 (8.0)	52	31	42	139.9 (22.7)	27.3 (4.5)	37.3 (12.3)
4b. Participated in T1, Phase I	6,468		59.3 (7.7)	53	37	31	136.7 (21.4)	27.0 (4.2)	41.5 (12.4)
5a. Not invited to T1, Phase II (cognition)		1,172	53.5 (8.5)	42	45	28	132.1 (20.7)	27.0 (4.3)	38.3 (9.8)
5b. Invited to T1, Phase II (cognition)	5,296		60.6 (6.9)	55	36	32	137.7 (21.4)	27.0 (4.1)	41.5 (12.4)
6a. Did not participate in T1 Phase II		783	61.8 (7.3)	53	32	37	140.0 (22.8)	26.9 (4.2)	39.0 (13.0)
6b. Participated in T1 Phase II	4,513		60.4 (6.8)	55	37	31	137.3 (21.1)	27.0 (4.1)	41.9 (12.3)
7a. Died before T1 data collection was finished		17							
7b. Alive when T1 data collection was finished	4,496		60.3 (6.8)	55	37	31	137.2 (21.1)	27.0 (4.1)	41.9 (12.3)
8a. T0 BP measurements out of range^∗^		31							
8b. T0 BP measurements within range	4,465								
9. Final Study Sample	4,465		60.3 (6.8)	55	37	31	136.8 (20.3)	27.0 (4.1)	42.0 (12.3)

* SBP > 200 mmHg, SBP < 80 mmHg, DBP > 120 mmHg, DBP < 30 mmHg. ** Available only for a subsample of 53%.

Every person in Tromsø aged 60–74 was invited to join our sample, together with a 40% random sample of those inhabitants younger than 60 years. Thus, invitations were sent to 12,054 Tromsø inhabitants aged 45–74, 8,813 of whom participated (Table 1). At T1, those 8,363 still alive and residing in Tromsø were contacted again for participation in T1, and 6,468 agreed to participate. Of this group of 6,468, a subset of 5,296 was invited to participate in a clinical examination including cognitive assessment, and 4,465 participated. Thus, our study sample comprised 51% of the baseline participants (4,465/8,813).

Our sample size was reduced because of a combination of missing random participants and those who dropped out due to poor health. In addition, 5% of the initial sample is missing because of death (433 died before attending T1) Among the 8,363 invited to T0, 1,895 or 22% of the initial sample did not agree to participate. This group differed from the participating group in that they were older, had less education, had higher baseline BP, higher baseline BMI, poorer baseline self-reported health, and had lower baseline cognitive scores (Table 1). Another large group of T1 participants (*n* = 1,172) were omitted from cognitive assessment at T1 because of the sampling design; fewer members of this group were women, and group members were substantially younger than those who were invited for cognitive assessment, and they had lower prevalence of poor self-reported health (Table 1).

Our final study sample (Table 1, item number 9) was slightly older (60.3 years vs. 59.9 years) than the entire baseline population (Table 1, item number 1b), consisted of more women (55% vs. 52%), were slightly more educated (37% vs. 36%), had lower BP at T0 (SBP = 136.8 mmHg vs. 137.7 mmHg), had lower prevalence of poor self-rated health (31% vs. 35%), and had better cognition at T0 (42.0 vs. 40.3 on the Digit Symbol Test of Wechsler Adult Intelligence Scale (WAIS). BMI at T0 was comparable in the two populations (27.0 kg/m^2^ vs. 27.1 kg/m^2^).

### Blood Pressure Measurements

Trained health personnel measured each participant’s BP on the upper right arm with a sized cuff based on arm circumference at the health survey. At both T0 and T1, BP was measured three times with an oscillometric digital automatic device: Dinamap ProCare 300 monitor, GE Healthcare, Norway. The measurements were separated by a 1-min interval after a 2-min seated rest, and the mean of the two final readings used in the analysis. (BP was modeled as a continuous variable in the analyses). BP level at T0, but not at T1, was regressed against cognition at T1, as we wanted to investigate how baseline BP affected later cognition. Nevertheless, BP at T1 was included for descriptive purposes, in order to facilitate a longitudinal pattern and change in BP from T0 to T1 for each birth cohort.

### Cognitive Tests

The following cognitive tests were applied: The Mini Mental State Examination (MMSE) (Folstein et al., 1975), the WAIS, Digit Symbol Test (Wechsler, 1955, 1967), and the Twelve-word Test (Rogne et al., 2013a) (a modification of the California Verbal Learning Test). The MMSE is a well-known screening test for dementia. The test includes orientation, attention, memory, language and visual-spatial skills. It includes 11 questions and takes 7–10 min to administer.

The MMSE is divided into two sections, the first based on vocal responses from the examinee, and covers orientation, memory, and attention; the maximum score is 21. The second part covers the ability to name, follow verbal and written commands, write a sentence spontaneously, and copy a polygon figure. The maximum score on the second part is nine. The total maximum score is 30.

In the Digit Symbol, a subtest from the Wechsler Adult Intelligence Scale (WAIS), the examinee is shown a series of symbols that are paired with numbers from 1 to 9. Using a key, the examinee is asked to consecutively fill in as many as possible of the blank spaces with the corresponding symbol as quickly and accurately as possibly whereby he or she draws each symbol under its corresponding number, within a 90 s. The score is the correct filled in symbols within the time limit. Participants were instructed not to skip symbols. Of all the Wechsler tests, the Digit Symbol Test is considered the most sensitive test of brain damage, even when the damage is minimal (Hirschenfang, 1960; Lezak, 1995); it is regarded as a test of processing speed and attention.

The 12 word memory test examine short-time verbal memory with immediate free recall of 12 nouns shown written on a board and also pronounced one at a time with a 5 s interval. The participants then had 2 min to recall the words, and one point was given for each word correctly recalled, and should therefore be able to discover memory difficulties (Palmer et al., 2003). The above were the reasons for choosing the tests in the Tromsø study (Rogne et al., 2013b; Jorde et al., 2015).

All together there were 10 study nurses, trained by either a specialist in geriatric diseases or a neuropsychologist at T0 and T1 respectively, who administered the tests. The tests were administered on the second visit (of two) as a part of the health examination in the Tromsø study, in especially dedicated study facilities.

### Covariates at Baseline

Self-reported use of antihypertensive drugs (*ever used* vs. *never used*) was included in the regression analyses as a covariate. Of the total participants, 80 (1.8%) had terminated their medication, and they were grouped as *ever users*. Education was grouped as low (corresponding to 9 years), medium (10–12 years), or high (13+ years). BMI (kg/m^2^) was included as a categorical variable (<20, 20–24, 25–29, 30+), and age was included as a continuous variable. Self-reported history of stroke was reported at both T0 and T1 (yes/no) and those reporting yes (*n* = 207) were omitted in a sensitivity analysis. Poor self-rated health was included as a dichotomous variable (1 = Very bad/bad/neither good nor bad, 0 = Good/Excellent).

### Statistical Methods

Stata/SE 15.0 was used for all analyses (StataCorp LLC). First, age trends in BP at T0 and T1 were investigated in linear regression, and gender differences in trends were investigated including age by gender interactions. Secondly, the association between baseline BP and cognitive function at follow-up was investigated with linear regression, adjusted by age, sex, BP medication use, education, poor self-rated health, and BMI. All two- and three-way interaction terms among BP, age, and sex were included. To visualize the results of the complex regression model, the *post-hoc* margins and marginsplot commands in Stata were applied. By the use of these commands, we provided estimates of the BP–cognition slopes at specified ages for men and women and could test whether slopes were significantly different from zero (no association). Statistical significance was defined as *p* ≤ 5%. In some settings we also showed *p* ≤ 10%, to indicate values near the significance level of 5%. The project was approved by the Regional Committees for Medical and Health Research Ethics (2016/389 REK sør-øst B).

## Results

### Blood Pressure by Age, Birth Cohort, and Sex

SBP increased with age (cross-sectional) at both T0 and T1 (Table 2). Women had lower SBP than men in the youngest age groups (<65 years), whereas women had higher SBP than men in the oldest age group (65–74 years). Thus, cross-sectionally, women’s SBP across age had a steeper increase than men’s SBP both at T0 and T1. When we examined birth cohorts longitudinally, we noted a trend toward *lower* SBP from T0 to T1 in men, despite their 8-year age increase. This tendency was present for all birth cohorts, but was most pronounced for the oldest groups (>54 years at T0). In women, however, the SBP increased over time, especially in the younger group, 45–54 years at T0.

**TABLE 2:** Background table.

Birth cohort	Age T0 (2007/2008)	Age T1 (2015/2016)	N	T0	T1	Change from T0 to T1
**Men**				**Systolic blood pressure**		

1953–1962	45–54	53–62	420	131.4 (16.2)	131.9 (16.9)	0.5 (16.4)
1943–1952	55–64	63–72	1,001	138.2 (18.6)	136.3 (18.2)	−1.8(18.3)
1933–1942	65–74	73–82	577	143.7 (19.3)	140.7 (18.8)	−3.0(20.1)
**Women**						
1953–1962	45–54	53–62	585	123.9 (18.2)	125.9 (17.7)	2.1 (15.9)
1943–1952	55–64	63–72	1,217	135.5 (19.9)	136.5 (19.9)	1.0 (19.8)
1933–1942	65–74	73–82	665	145.7 (21.2)	146.2 (21.3)	0.5 (23.3)

**Men**				**Diastolic blood pressure**		

1953–1962	45–54	53–62	420	82.2 (9.7)	79.4 (8.9)	−2.8(9.1)
1943–1952	55–64	63–72	1,001	82.6 (10.1)	77.6 (9.7)	−5.0(9.8)
1933–1942	65–74	73–82	577	80.7 (9.4)	75.7 (9.4)	−5.0(10.1)
**Women**						
1953–1962	45–54	53–62	585	75.0 (9.8)	73.9 (9.4)	−1.1(8.6)
1943–1952	55–64	63–72	1,217	75.9 (9.6)	73.6 (9.4)	−2.3(9.1)
1933–1942	65–74	73–82	665	75.2 (9.7)	73.4 (9.7)	−1.8(10.3)

**BP at baseline in Tromsø Wave 6 (T0) and at follow-up in Tromsø Wave 7 (T1) and change. N* = 4,465.*

### Cognition by Age, Sex, and Education

Overall, cognitive performance declined with age, at a similar pace for men and women, on all three cognitive measures. Scores on the Digit Symbol Test declined by 0.7 units per year (*p* < 0.001), and scores on both MMSE and the Twelve-word Test declined by 0.07 units per year (*p* < 0.001). Men had significantly (*p* < 0.001) poorer performance than women did on all three cognitive tests, adjusted by age and education. Men’s scores averaged 4.2 points lower than women’s scores on the Digit Symbol Test, 0.4 lower on the MMSE, and 0.6 units lower on the Twelve-word Test. Education was significantly associated with better cognitive performance on all three tests, in a step-wise pattern (adjusted by age and sex). Participants with higher education scored 8.4 points higher on the Digit Symbol Test than did participants with less education, 1.4 higher on the MMSE, and 1.3 higher on the Twelve-word Test (all tests *p* < 0.001).

### The Relationship Between Baseline BP and Cognition at Follow-Up

The association between baseline BP and cognition at follow-up was related to both age and sex. Furthermore, results were not consistent across the three cognitive outcome measures, with the Digit Symbol Test most strongly associated with BP. There was a three-way interaction term for this measure: BP by age by sex was significant for SBP (*p* = 0.005) and DBP (*p* = 0.005).

#### Results for Men

**FIGURE 1:**
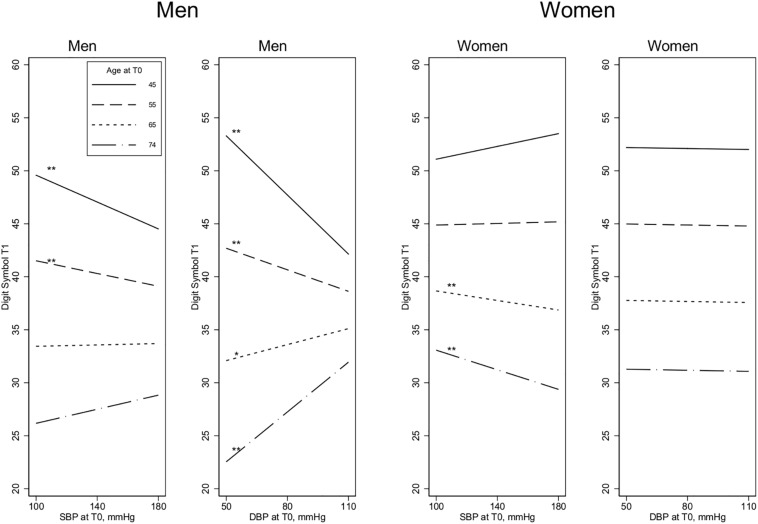
Digit Symbol Test at T1 and the association with BP measurements at T0. Age is measured at T0, 8 years before the cognitive assessment at T1. SBP, Systolic blood pressure; DBP, Diastolic blood pressure. Estimated in linear regression, adjusted by baseline covariates [BP medication, education, self-reported health, and body mass index (BMI)]. All the 2-way and 3-way interaction terms – blood pressure by age by sex – are included. Test of slope different from zero: *p < 0.1, **p < 0.05.

**FIGURE 2:**
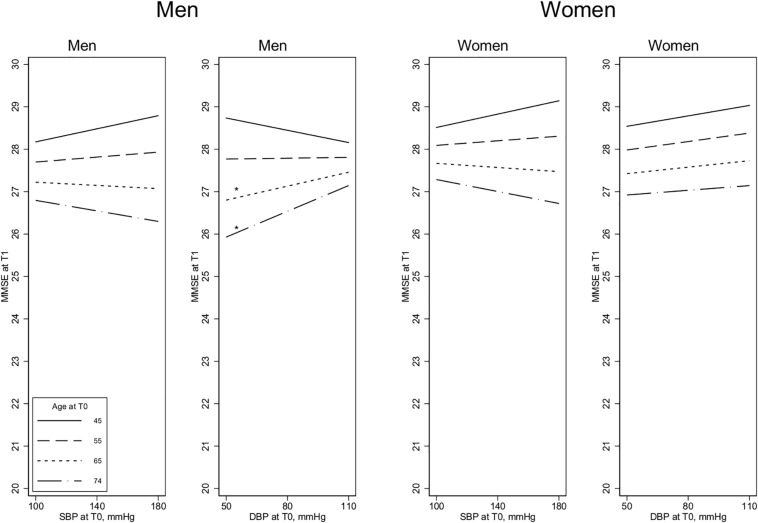
MMSE scores at T1 and the association with blood pressure measurements at T0. Age is measured at T0, 8 years before the cognitive assessment at T1. SBP, Systolic blood pressure; DBP, Diastolic blood pressure. Estimated in linear regression, adjusted by baseline covariates [BP medication, education, self-reported health, and body mass index (BMI)]. All the 2-way and 3-way interaction terms – blood pressure by age by sex – are included. Test of slope different from zero: *p < 0.1, **p < 0.05.

**FIGURE 3:**
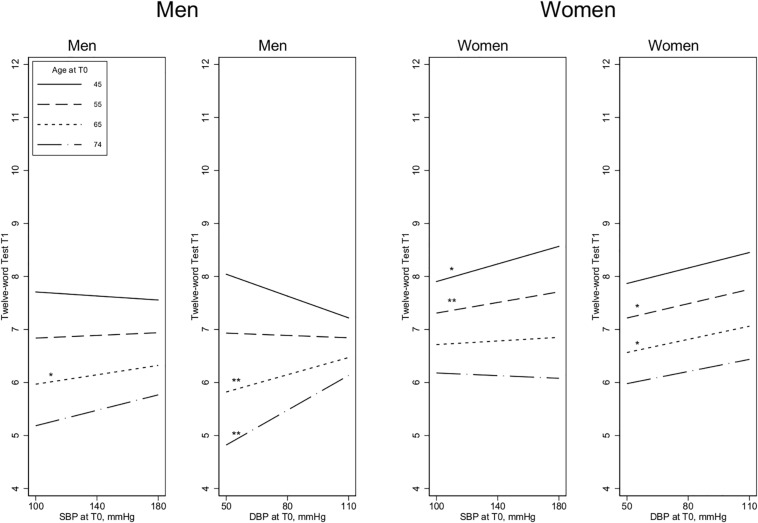
The Twelve-word Test scores at T1 and the association with blood pressure measurements at T0. Age is measured at T0, 8 years before the cognitive assessment at T1. SBP, Systolic blood pressure; DBP, Diastolic blood pressure. Estimated in linear regression, adjusted by baseline covariates [BP medication, education, self-reported health, and body mass index (BMI)]. All the 2-way and 3-way interaction terms – blood pressure by age by sex – are included. Test of slope different from zero: *p < 0.1, **p < 0.05.

In younger men (45–55 years), higher SBP and DBP levels were significantly associated with *poorer* performance on the Digit Symbol Test at the 8-year follow-up, whereas higher BP among older participants (65–74 years) was associated with *better* cognition at follow-up, especially for DBP (age by SBP interaction term: *p* = 0.039; age by DBP interaction term: *p* < 0.001) (Figure 1). For the other two cognitive measures (MMSE and Twelve-word Test), the associations with SBP were weaker, and the age by SBP interaction terms were not significant for either measure (Figures 2, 3). For DBP, however, similar age patterns were observed for the other cognitive measures as for the Digit Symbol Test, with a significant age by DBP interaction term for the Twelve-word Test (*p* = 0.043) and a non-significant association for MMSE (*p* = 0.100).

#### Results for Women

Contrary to the findings for men, women’s higher SBP in the older age groups (65–74 years) was associated with significantly *poorer* performance on the Digit Symbol Test, although there was no significant association at younger ages (The age by SBP interaction term was not significant *p* = 0.051; see Figure 1.) For the other two cognitive measures, there were no significant age by SBP interactions. There were no significant DBP by age interactions, and the association between SBP and cognition was generally weak and non-significant in women, except for performance on the Twelve-word Test, in which higher DBP and higher SBP at younger ages (45–55 years) were associated with significantly better cognition (Figures 2, 3).

### Sensitivity Analyses

When we omitted the 207 (4.6%) participants with self-reported history of stroke at either T0 or T1, almost identical results were obtained, and did not change any of the main findings or conclusions.

## Discussion

In this large population-based health examination study, we found that for the youngest cohort of men (45–55 years of age), higher BP was associated with poorer cognitive performance 8 years later. This tendency was not seen for the women. For the oldest men (above 65), however, higher BP was associated with better cognition. For the oldest women, on the other hand, higher SBP was associated with poorer cognitive performance 8 years later, whereas in the younger cohort there were no clear trends between BP and cognition. Thus, we suggest a tendency for differences in association of elevated BP on cognitive performance between the two sexes. To our knowledge, this is the first time such a sex by BP interaction has been reported for cognition.

In our study, in accordance with a recent report from the Tromsø study (Hopstock et al., 2015), SBP was stable in women (actually a small increase in mean score) and falling with age in men.

The study by Hopstock et al. suggests that BP does not increase with age. However, some limitations were discussed, the major one being that 40% of subjects had only one BP measurement and there was not a complete follow-up of subjects. Thus, there might be some selection bias impacting the age trend in BP. Another study from Norway (Holmen et al., 2016) came to the same conclusion in a large population based study, namely that blood pressure levels decreased substantially from mid 1980s to mid 2000s. Even though the blood pressure medication increased this only explained a small part of the blood pressure reduction as the decrease was also considerable in those who reported to never use such a medication. The decrease was biggest in those who were in the oldest age group (80+). Our study, partially supports such a suggestion regarding blood pressure decrease with age especially regarding diastolic BP. Examination of the data cross sectional however, there are still large age differences.

There is clearly a difference between women and men in the way BP seems to relate to cognition in the aging process. Could it be related to their different trajectories regarding heart disease? Even though men and women experience the same risk factors for myocardial infarction, they demonstrate a different pattern in incidents of myocardial infarction (Barrett-Connor, 2013). An epidemiological study from the same cohort as our study have shown an accelerated myocardial infarction risk in young men compared to young women, which decreases as they age (Albrektsen et al., 2016). The incident rates increased with age in both sexes, with lower rates for women until 95 years of age. This gap between the sexes declined with age as a result of a more pronounced flattening of risk level changes in middle-aged men (Conroy et al., 2003; Barrett-Connor, 2013; Albrektsen et al., 2016; Selmer et al., 2017). Despite the decrease in incidence in older men, women never catch up (Albrektsen et al., 2016; Barrett-Connor, 2013). Women usually get cardiovascular disease 6–10 years later than men do, despite similar risk factors. Albrektsen et al. (2016) conclude that this observation in myocardial infarction cannot be explained by differences in established coronary heart disease risk factors. The different pattern between men and women may be explained by different development of blood pressure by biological aging, e.g., that women have longer life expectancy and thereby different development of blood pressure. As there is no acceleration in risk after menopause, hormonal factors cannot be solely responsible for the observed sex differences. But as women tend to increase more in BMI by age than men do, the estrogen produced by fatty tissue may still play a part in protecting women from cognitive dysfunction (Jacobsen et al., 2001). Even so, we cannot exclude the possibility that hormones, especially estrogen, can affect the brain and create the difference between the sexes regarding cognitive aging. For elderly women, postmenopausal hypertension, with changes in their estrogen/androgen ratio; activation of the Renin-angiotensin system, sympathetic nervous system, and the endothelin-1 system may result in more inflammation and increased vasoconstrictor eicosanoids (Song et al., 2019). These changes may influence sex differences in the aging process and in cognitive performance. There seems to be a link between cardiovascular health and cognition even before the development of dementia. We cannot rule out the possibility that some of the inferior cognition we see in our research is the beginning of Alzheimer’s disease as men also get Alzheimer at a younger age than women. The adverse effect of high BP on cognition among the youngest male cohort was most pronounced for the Digit Symbol Test (less so but to some degree, also for the Twelve-word Test and MMSE), which is no surprise, given that the Digit Symbol Test is highly sensitive to cognitive impairment. Our data indicate, as for studies related to myocardial infarctions, that the effect of elevated BP on cognition occurs later in life for women compared to men (significant BP × age × sex interaction). As for the MMSE it must be noted that all the mean scores are above 23, and that this test is sensitive to the development of dementia; the MMSE may be less sensitive to minor cognitive deficits, however. A score below 23 is seen as indicating possible dementia, and a score above this cutoff is considered normal or close to normal, depending on educational level and age (Folstein et al., 1975). Our results may suggest that increasing BP impairs cognition in men, but we cannot exclude the possibility of reverse causality: cognitive impairment (from possible cerebral affection) may create a drop in BP (Joas et al., 2012). The association between BP and cognition is more uncertain in the oldest group in our sample, because the associations point in different directions, and with less clear associations between BP and cognition. In a previous population-based study of people above the age of 80 years, we showed that low BP was highly associated with low scores on the MMSE (Hestad et al., 2005; Hestad and Engedal, 2006). In the present study, however, the older women were more like the younger men, as high SBP was associated with poor cognitive performance.

We did not observe any influence of the use of anti-hypertensive drugs. Perhaps the analyses were unable to unearth the benefits of the drugs with regard to performance on cognitive tests. Alternatively, the brain damage related to high BP before the treatment with medication may not have been ameliorated, especially given that more than 60% of the participants on treatment were above treatment target. There are conflicting results from other studies related to antihypertensive therapy and the effects on cognitive functioning. Although the McGuinness et al. (2009) meta-analysis found no association between the lowering of BP and cognitive impairment, Tzourio et al. (2003) found that reducing BP had a positive effect on cognition – but only by reducing the rate of stroke. A recent randomized clinical trial demonstrated that intensive SBP control can reduce the development of mild cognitive impairment (Williamson et al., 2019).

Our sensitivity analyses indicated that those with the lowest and highest BP at baseline dropped out of the study at follow-up, which may indicate that those with the worst prognosis regarding cognition were not included in the analyses. Dropout from longitudinal population-based studies usually reflects sickness or poorer functioning among dropouts than among those who remain in the study. The dropout rate could therefore reflect that both those with a low and high pressure have developed a worsening of their situation, and perhaps cognitive decline and dementia.

The participants in this study were all volunteers, and supposedly no one had dementia – at least no one was diagnosed with dementia.

One strength of our study was the large number of participants. Few researchers have had the opportunity to examine change in BP related to cognition in more than 4,000 participants.

A possible limitation in our study is selection bias due to selective drop out from T0 to T1. Those participating in both T1 and T0 differed from those who only participated in T0 in that they had better self-reported health and cognition at T0. They were also younger, had higher education, lower baseline BP, and lower BMI. These biases were addressed by adjustment of factors associated with the dropouts. However, there may still be some bias that may have influenced findings and thus our conclusions. This bias would likely “mute” any true associations between BP and cognition. Four hundred and sixteen participants (4.7%) were not invited to T1 because they had emigrated or died before follow-up. Of these 60% were men and 40% women. The observed longitudinal decrease in BP in men, may to some degree be due to a higher mortality compared to women with only survivors with a lower BP remaining. Morbidity might be a confounding factor, as well as a factor related to drop out between T0 and T1. Unfortunately, we did not have morbidity information, except from stroke history, and had to rely on self-reported health status.

Another limitation regarding the difference between men and women and the delay observed in these latter, may be due to the fact that many of the younger women are still in the premenopausal phase, even at the end of 8 years of follow-up. In this phase of life, women may be at a lower risk of dementia and cardio vascular events.

A third limitation of study was the fact that only three cognitive tests were used. The Digit Symbol Test, which is related to processing speed and attention, is considered to be a highly sensitive test for brain damage, and the results of this test performance were consistently affected primarily by high BP, but also by low BP. The Twelve-word Test showed some association with BP. We did not have any delayed recall performance, but in populations such as these, the correlation between immediate and delayed recall is usually high (Nespollo et al., 2017).

Some of the earlier studies related to BP and dementia have examined only women or only men. Both sexes were represented in our study, no diagnosis of dementia was present before testing, and we found a different trend in BP across age and sex.

In conclusion, high BP at baseline for the men, but not the women, was clearly associated with reduced cognitive function 8 years later. Because it may be that in some brain disorders, like small-vessel disease or Alzheimer’s disease, brain damage can cause both cognitive deficits and a decrease in BP, we cannot claim to have found a causal relationship, however.

## Data Availability Statement

The datasets for this article will not be made publicly available because: There are legal restrictions, imposed by the data owner and the ethical committee, on sharing our de-identified data set due to sensitive information. Only approved project members are allowed access to the data. Contact information for the ethical committee and the data owner: (1) Regional Committees for Medical and Health Research Ethics (REC), post@helseforskning.etikkom.no; (2) The Tromsø Study, tromsous@uit.no

## Ethics Statement

The studies involving human participants were reviewed and approved by Regional Committees for Medical and Health Research Ethics (REC) (2016/389 REK sør-øst B). The patients/participants provided their written informed consent to participate in this study.

## Author Contributions

KH initiated and planned the study. HS supervised the blood pressure content. KE took part in the planning of the study. BS did all the statistical analyzes. KH and BS wrote the first draft of the manuscript. All authors took part in the writing process.

## Conflict of Interest

The authors declare that the research was conducted in the absence of any commercial or financial relationships that could be construed as a potential conflict of interest.

## Footnotes

1 www.tromsoundersokelsen.no
